# In Extenuation

**Published:** 1887-09

**Authors:** 


### IN EXTENUATION.

It is a source of regret that a report of the June clinic of the Chicago Dental Club is unavoidably crowded out of this number. Other reports and articles had precedence. An excellent paper by Dr. W. H. Trueman, read before the Central Dental Association of Northern New Jersey, with the discussion upon it, is also put over a month. We cannot accomplish the impossible, and ask that our good friends will extend forbearance.

